# Impact Value Improvement of Polycarbonate by Addition of Layered Carbon Fiber Reinforcement and Effect of Electron Beam Treatment

**DOI:** 10.3390/polym17081034

**Published:** 2025-04-11

**Authors:** Yoshitake Nishi, Naruya Tsuyuki, Michael C. Faudree, Helmut Takahiro Uchida, Kouhei Sagawa, Yoshihito Matsumura, Michelle Salvia, Hideki Kimura

**Affiliations:** 1 Graduate School of Engineering, Tokai University, Hiratsuka 259-1292, Japan; west@tokai.ac.jp (Y.N.); 6bajm036@mail.u-tokai.ac.jp (N.T.); helmutuchida@tokai.ac.jp (H.T.U.); sagawa.kouhei@tokai.ac.jp (K.S.); ncc1701d@keyaki.cc.u-tokai.ac.jp (Y.M.); kimura@tokai-u.jp (H.K.); 2 Graduate School of Science & Technology, Tokai University, Hiratsuka 259-1292, Japan; 3 Laboratoire de Génie Electrique et Ferroéléctricité (LGEF), INSA Lyon, CEDEX, 69621 Villeurbanne, France; 4 Ecole Centrale de Lyon, CEDEX, 69134 Ecully, France; michelle.salvia@ec-lyon.fr; 5 Kanagawa Institute of Industrial Science and Technology (KISTEC), Ebina 243-0435, Japan; 6 Faculty of Liberal Arts and Science, Tokyo City University, Yokohama 224-8551, Japan

**Keywords:** composite, recyclable thermoplastic, polycarbonate, carbon fiber, electron beam, impact value

## Abstract

Polycarbonate (PC) is a highly recyclable thermoplastic with high impact strength that bodes well to re-melting extrusion and shredding for positive environmental impact. For the goal of improving impact strength further, layered carbon fiber (CF) reinforcement has been added between PC sheets by hot pressing at 6.0 MPa and 537 K for 8 min. An addition of cross-weave CF layer reinforcement to PC increased Charpy impact value, *a*_uc_ of the untreated [PC]_4_[CF]_3_ composite over that of untreated PC resin reported at all accumulative probabilities, *P*_f_. At medial-*P*_f_ of 0.50, *a*_uc_ was increased 3.13 times (213%), while statistically lowest impact value *a*_s_ at *P*_f_ = 0 calculated by 3-parameter Weibull equation was boosted 2.64 times (164%). To optimize *a*_uc_, effect of homogeneous electron beam irradiation (HLEBI) treatment of 43.2, 129, 216, 302, or 432 kGy at 170 kV acceleration voltage to the CF plies before assembly with PC then hot press was also investigated. The 216 kGy HLEBI dose appears to be optimum, raising *a*_s_ at *P*_f_ = 0 about 6.5% over that of untreated [PC]_4_[CF]_3_ and agrees with a previous study that showed 216 kGy to be optimum for static 3-point bending strength, when quality can be controlled. Electron spin resonance (ESR) data confirms 216 kGy HLEBI generates strong peaks in CF and PC indicating dangling bond (DB) generation. Bending strength increase was higher than that of impact due to lower test velocity and higher deformation area spreading along specimen length, allowing more DBs to take on the load. X-ray photoelectron spectroscopy (XPS) data of CF top ~10 nm surface layer in the sizing confirms C–O–H, C–H, and C–O peak height from 216 kGy exhibited little or no change compared with untreated. However, 432 kGy increased the peak heights indicating enhanced adhesion to PC. However, 432 kGy degraded *a*_s_ at *P*_f_ = 0 of the [PC]_4_[CF]_3_, and is reported to decrease impact strength of PC itself by excess dangling bond formation. Thus, the 432 kGy created increased PC/CF adhesion, but degraded the PC resin. Therefore, 216 kGy of 170 kV-HLEBI appeared to be a well-balanced condition between the PC-cohesive force and PC/CF interface adhesive force when fabricating [PC]_4_[CF]_3_.

## 1. Introduction

Single-use plastics pose a serious threat to our Earth’s environment. A 2022 report by the Organisation for Economic Co-operation and Development (OECD) states that plastic pollution is increasing relentlessly while recycling and waste management policies fall short of those required for a sustainable eco-system [1]. Globally, only 9% is successfully recycled, while 49% is carelessly thrown into landfills, 19% is incinerated, and 22% is littered directly into the environment [1]. The careless throwing away of plastic has been polluting our lands, waters, and atmosphere. Polycarbonate (PC), on the other hand, is a thermoplastic (TP) that is highly recyclable that can be repeatedly re-melted and reformed when parts become unusable. Energy consumption for solidification is about 10% that of non-recyclable epoxy thermoset (TS) polymers. Since the short production cycle of TPs advances cost reduction, glass fiber reinforced thermoplastic polymers (GFRTPs) [2,3] can be generally applied to automobiles. PC bodes well to re-melting extrusion and shredding, and the scrap can be melted and reformed for new parts to minimize loss of material. A drawback, however, is that most TPs are extracted from petroleum. However, with recyclability, it is possible the need to extract additional petroleum resources from the Earth can be reduced or eliminated. This helps TPs to be good candidates to use in future settlements in space such as on the moon and on Mars.

**Figure 1:**
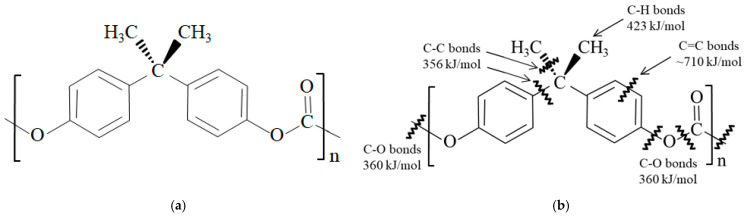
Constitutional formula of (**a**) PC monomer, and (**b**) that with bonding dissociation energies [5,6].

PC depicted in Figure 1a, is widely used for numerous articles, in particular for those requiring optical clarity such as aircraft windows, vehicle headlights, bulletproof glass, and safety goggles [4]. The lightweight property of PC allows it to be used for aerospace and land transport vehicles for various molded parts such as coverings and casings. PC also finds decent use for laptop computer and cell phone cases due to its crack resistance.

The wide use of PC has been due to several merits [4,7,8]. PC has high impact resistance, and can lower fuel consumption and CO_2_ emissions, and has a high strength-to-weight ratio, hence it is a key candidate for use in various vehicles. In addition, PC has a high glass transition temperature (*T*_g_) of 154 °C (428 K) [7] to withstand higher temperatures without melting or deforming. PC allows superior electrical insulation materials often utilized for electronics for prevention of short circuits or other electrical hazards. A drawback of PC, in addition to typically having to be extracted from petroleum, is non-suitability for food contact since effusion of bisphenol-A (BPA) can absorb into food. However, BPA-free formulations have been fabricated [4]. Although PC has good ultraviolet ray (UVR) resistance, longer UV exposure can induce discoloration and loss of strength [4]. Moreover, to overcome its lower flame resistance, additives must be used to meet fire safety standards for maximum safety; however, caution is needed because additives can be toxic.

However, for thermoplastic (TP) CFRTPs, a main drawback has been significantly lower mechanical strength compared with CFRP epoxies due to poor adhesion at the TP/CF interface. Both TP and CF have chemically inert surfaces with non-polar molecular structure, along with low-wettability, and hydrophobicity [9]. Moreover, smoothness of the CF surface can also lead to insufficient adhesion. 

Hence, there have been numerous methods to strengthen CFRPs at the CF/Polymer interface [10,11,12,13,14,15,16,17,18,19,20,21,22,23,24,25,26,27,28,29,30,31,32,33,34,35,36,37,38,39,40,41,42]. Modifying CF sizing has been a particular focus [10,11,12,13,14] since most CF used is coated with epoxy or other sizing. Other studies have investigated acidic modification to create higher interfacial friction at the CF surface by [15]; however, drawbacks are lowered strength [16,17] by degradation of the surface, along with weight reduction of the fiber itself [18]. Superheated steam for 1 h at 650 °C (K) has been used on recycled CF to increase adhesion to polypropylene (PP) resin by the addition of functional groups containing oxygen [19]. CF surface modifications by applying plasma have been extensively researched [20,21,22] and have been found to enhance CFRP interlaminar shear strength. Interfacial shear stress in epoxy CFRP is reported to be increased by about 7 times by plasma oxidation treatment to the CF surface [23]. Since CF and TP surfaces are nonpolar, several studies have focused on creating polar groups on the CF surface [24,25,26], coating with electro-polymer [24] attachment of rare earth particles [25,26], and high energy irradiation treatments [27,28], such as Ar+ [29] and Co^60^ γ-ray [30]. Other studies have investigated applying multi-walled nanotubes with copper (Cu) (MWNT/Cu) to the CF surface [31,32]. Also, chemical treatment to the CF surface has been receiving ample attention, including ozone (O_3_) [33,34], anodization [35,36,37], hydrogen peroxide (H_2_O_2_) in supercritical H_2_O [38], aqueous ammonia [39], nitric acid (HNO_3_) [40,41], and oxy fluorination [42].

For CFRTP composites, several studies of strengthening CFRPCs at the CF/TP interface exist in the literature [8,43,44,45,46,47,48,49,50,51,52,53]. These include sandwich structures using PC core between two plies of CFRP (CFRP/PC/CFRP) that were found to exhibit higher Charpy impact strength than CFRPC itself to lower material cost [43]; lowering viscosity of PC to decrease void size and successfully impregnate CFs in CFRPC [8]; and a novel 3D printing process of manual placement of CF bundles with long CFs achieving CFRPC with 0% porosity [44]. Other findings are as follows: hot pressed CFRPC had a 46% to 81% increase in interfacial shear strength (IFSS) over that of solution cast [45]; and CFRPC with sized CFs exhibited higher tensile strength and modulus than that of unsized [46]. As for high radiation resistance, CFRPC subjected to a γ-ray environment at 737 Gyh^−1^ dose rate was reported to be safe until it reached a 75 kGy dose [47]. For the CF coatings, a combination of spray coating, and longer hot press time was found to raise IFSS. Optimum coating thickness was found to be between 0.15 and 0.32 μm [48]. Moreover, a controlled process of pre-coating CFs with PC nanoparticles (PC-NPs) was found to improve PC impregnation to raise shear, tensile, and flexural strengths 62%, 41% and 22%, respectively, [49]. Another method that has found success is polymer modification of the PC resin itself with ethyl-3,5-dihydrooxybenzoate to act as reactive sequences in the PC backbone, increasing PC/CF adhesion as evidenced by single CF test in pressed films [50]. Coating the CF surface with carbon nanotubes (CNTs) has found success in strengthening the PC/CF interface [50,51,52,53]. A combination of an electrophoteric deposition process (EDP) of CNT on the CF surface to increase contact surface area, with a solution impregnation pre-treatment (SIP) has been carried out [51]. Results showed the fabricated CF/CNT/PC was strengthened significantly over that of CFRPC (without CNT) with tensile strength, modulus, impact strength, and storage modulus raised 46.5%, 57.5%, 268.7%, and 78.4%, respectively, to 344.8 MPa, 37.8 GPa, 36.5 kJm^−2^, and 49.0 GPa [51]. Improvements were attributed to allowing PC to exhibit high contact area and impregnation with the CNT coated CF [51]. In addition, CNT to fabricate CF/CNT/PC was found to increase PC/CF adhesion along with thermal transfer under short beam testing [52]. In a plasma-treated CF/CNT/PC composite, storage modulus and absorbed impact energy were increased by 387% and 194% over that of untreated CFRPC [53].

On the other hand, homogeneous low voltage electron beam irradiation (HLEBI) does not use any additives, chemicals, or particles, has shown success in raising mechanical properties of CFRP composites [54,55], and has the benefits of low cost, clean operation, and possibility of use at room temperature [56]. Th electron beam method has been used in industry for polymerization, crosslinking, and processing without an initiator [57]. When HLEBI is applied to both outside surfaces of finished samples of CFRP with thermo-hardened epoxy matrix after fabrication, it raised Charpy impact strength [55], and at low fracture probability, *P*_f_ increased Charpy impact strength 56% in thermoplastic PEEK (Polyetheretherketone) CFRP samples, increasing reliability and safety of the weakest samples in the data set [54].

HLEBI is a low-energy electron curtain beam that penetrates into the specimen surface, utilized to strengthen materials by generating dangling bonds (DBs) [58], i.e., severing bonds [59]. In polymers, DBs are in the form of nano-compressive sites, where lone pair electrons repulse each other to increase strength in the polymers themselves, as well as creating strong TP/CF bonds at the interface [59]. As shown in Figure 1b for PC, DBs are generated at bonds with low bond dissociation energy (BDE), namely, C–C (356 kJ/mol) and C–O bonds (360 kJ/mol), respectively [5,6]. On the other hand, BDE of H–C=C–H (~710 kJ/mol) and C–H bonds (423 kJ/mol) are significantly higher; thus, the probability of DB generation is lower than those of C–C and C–O.

With the goal of increasing sustainability and recyclability, HLEBI has shown success in strengthening CFRTP interlayered structures to overcome the poor adhesion at the TP/CF interface [60,61,62,63,64,65,66]. In 3D printed short CF polyamide 66 (3D-CFRPA66), applying a HLEBI dose of 43.2 kGy at acceleration potential, *V*_c_ of 210 kV, tensile elasticity and a measure of toughness were apparently increased 771% and 504%, respectively, over that of untreated [60]. However, the tradeoff was that tensile strength was reduced by 50%, indicating mechanical properties can be tailored according to application. But strength needs to be balanced with ductility for material consolidation and ease of processing [61]. In a hot-pressed CFR-Polyamide (CFRPA) layered structure, impact strength was increased by applying 43.2 kGy HLEBI to finished samples with *V*_c_ set at 250 kV [62]. For CFRTP polyphenylene sulfide (CFRTP-PPS) interlayered composite with [PPS]_4_[CF]_3_ layup with alternating PPS and CF, applying a low dose of 5 kGy HLEBI at *V*_c_ of 170 kV to PPS plies prior to lamination assembly and hot press increased average impact strength 16% from 20.4 to 23.8 kJm^−2^, along with statistically lowest impact value for design, (*a*_s_ at *P*_f_ = 0) calculated by 3-parameter Weibull analysis, from 0 to 19.9 kJm^−2^ [63]. Moreover, for interlayered polypropylene composite, [PP]_4_[CF]_3_, applying 129 kGy HLEBI to CF plies prior to lamination assembly and hot press raised impact values at all fracture probabilities, *P*_f_. Here, an impact value at low-*P*_f_ = 0.07 was increased by about 103%, with *a*_s_ at *P*_f_ = 0 improved by 110% [64]. Bending strength, *σ*_b_ of interlayered composite with removed sizing film (sizing film free) [PP]_4_[SFF-CF]_3_ has also been reported to be increased at all *P*_f_ by 0.22 MGy EBI under 2000 ppm O_2_ rich atmosphere [65]. At low, median, and high *P*_f_, of 0.06, 0.50, and 0.94, the *σ*_b_ were apparently improved by 23%, 26%, and 20% over that of untreated. And for the material in this study, [PC]_4_[CF]_3_, it was previously reported when 216 kGy HLEBI was applied to both sides of CF plies before lamination assembly and hot press, static 3-point bending strength, *σ*_b_ of an 11-sample data set was increased above untreated at all *P*_f_. When quality could be controlled by eliminating the weakest sample in the data set, *σ*_b_ at median-*P*_f_ of 0.50 of the 10-sample data set was increased by 25% over that of untreated CF [66].

Unexpectedly, this study of the [PC]_4_[CF]_3_ composite is congruent with the bending study [66]: 216 kGy HLEBI directly to CF plies before assembly and hot press appears to be the optimum dose for impact strength. To explain the rationale of choosing 216 kGy, other HLEBI doses 42.3, 129, 302, and 432 kGy yielded smaller impact values for safety. In the literature, there are very few studies found of electron beam to CFRPC, while those on effects of electron beam to PC resin on mechanical properties are sparse [67]. Chen et al. (2005) found that for PC samples, applying high voltage 2 MeV electron beam irradiation with increasing doses of 25, 50, 100, 150, or 200 kGy decreased tensile strength and elongation successively from about ~65.7 to ~63.2 MPa, and ~74 to ~51%, respectively [67]. However, it was found that when applying the low voltage 170 keV HLEBI of 129 kGy homogeneously, mechanical properties can be increased: average impact strength of PC resin was increased by 25% from 25.5 to 33.8 kJm^−2^ [68]. Other studies of electron beam to PC are limited to investigating physical properties including surface characterization of PC films indicating cross-linking density increase at the surface [69], high voltage electron beam to PC films to characterize dosimetric properties’ [70] effect on physio-mechanical properties [71], structural [72] and thermal properties [72,73], and optical and electrical properties [74].

Therefore, to fill the research gap, we investigate the effects of HLEBI directly to CF plies on impact strength of an interlayered [PC]_4_[CF]_3_ composite. The goals of this research are two-fold. First, we demonstrate that the high impact strength of PC resin can be raised 2 to 3 times by adding interlayered carbon fiber (CF) plies between PC sheets by hot press. Second, we show for the resulting CFRTP, impact strength can be increased further by using homogeneous low voltage electron beam irradiation (HLEBI), increasing the strength of the weakest samples without the use of additives, chemicals, or particles. This is with the goal of developing strong composites that are recyclable and easy to process to achieve sustainable development goals (SDGs).

## 2. Materials and Methods

### 2.1. Composite Fabrication

**Figure 2:**
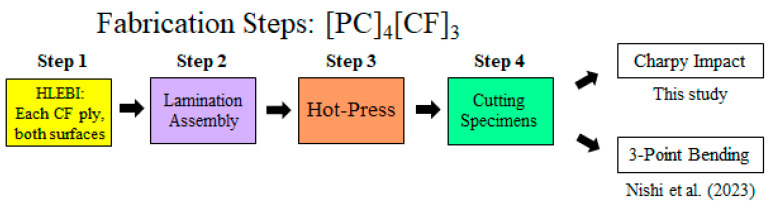
Fabrication steps of HLEBI-treated [PC]_4_[CF]_3_ interlayered composite for impact, and that reported for 3-point bending [66]. (For untreated, Step 1 is skipped).

The 55vol%-carbon fiber reinforced thermoplastic polycarbonate (CFRPC) samples were constructed with sized plain cross-weave CF plies (TR3110M, Mitsubishi Rayon Ltd., Tokyo, Japan) that were 230 μm thick before molding with an areal weight of 198 to 200 gm^−2^ [75]. TP PC sheets (110 mm × 170 mm × ~286 µm) (Sugawara Kougei Ltd., Tokyo, Japan) were fabricated by hot-pressing PC particles (3 g) under 15 MPa at 418 K for 3 min. Fabrication steps shown in Figure 2 were as follows: **Step 1** is each CF ply is treated with HLEBI on both side surfaces (see next section). **Step 2** is lamination assembly, where 3 CF plies were interlayered between 4 PC sheets, layer by layer, with [PC/CF/PC/CF/PC/CF/PC] layup, designated here as “[PC]_4_[CF]_3_”. **Step 3** is hot-press processing (IMC-185A, Imoto Machinery Co., Ltd., Tokyo, Japan) under 6.0 MPa and 537 K for 8 min. **Step 4** is cutting specimens into dimensions: length, width, and thickness of 80 × 10 × 2.0 mm according to Japanese Industrial Standard JIS K 7077 [76]. Only the CF plies were HLEBI treated; the PC plies were not treated.

### 2.2. Condition of HLEBI

**Figure 3:**
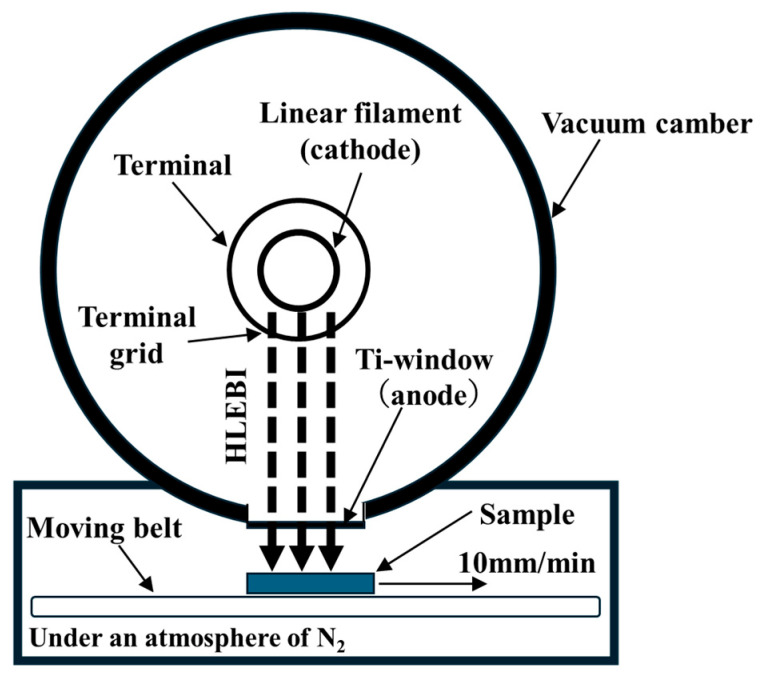
Schematic of electron beam curtain processor.

A test matrix consisting of untreated [PC]_4_[CF]_3_, along with those treated by HLEBI doses of 43.2, 129, 216, 302, or 432 kGy directly to the CF plies prior to lamination assembly and hot press was carried out. Dose levels were chosen to be congruent with a previous study on bending of [PC]_4_[CF]_3_ [66]. The CF plies were homogeneously treated on both sides by an electron-curtain processor (Type CB175/15/180L, Energy Science Inc., Woburn, MA, USA, Iwasaki Electric Group Co., Ltd., Tokyo, Japan) as illustrated in Figure 3 [63,64,65,66]. This was prior to layup and hot pressing the [PC]_4_[CF]_3_ laminate. Samples were homogeneously irradiated by the linear electron beam gun with low energy through a titanium thin film window attached to a 240 mm diameter vacuum chamber. The electron beam was generated by a tungsten filament in a vacuum at the low energy condition, with acceleration potential (i.e., cathode voltage, *V*_c_) of 170 keV with irradiating current density (*I*) of 0.089 A·m^−2^ for one operation time. Distance between sample and titanium window was 25 mm. Although the electron beam is generated in a vacuum, the irradiated sample was kept under protective N_2(gas)_ atmosphere with residual concentration of O_2(gas)_ less than 300 ppm. Flow of N_2(gas)_ was set to be constant at 1.5 L/s at 0.1 MPa N_2(gas)_ pressure. Single CF plies were wrapped in one layer of wax paper, placed in a 0.15 × 0.15 m aluminum plate holder and transported on a conveyor at constant speed of 10 m/min. The sheet electron beam irradiation was applied intermittently, sample tray was swept back and forth until total HLEBI dose was achieved. One sweep going one way was 43.2 kGy. Each 43.2 kGy irradiation dose was applied for only a short time (0.23 s) with 30 s interval between each sweep to avoid excessive heating of the sample. Sample surface temperatures remained below 323 K just after irradiation. Samples were then turned over and the other side administered the same HLEBI dose. Irradiated dose is proportional to irradiation current (*I*, mA) and number of irradiations (*N*), and inversely proportional to the conveyor speed (*S*, m/min).

Irradiation dose was controlled by integrated irradiation time in each sample. Here, the irradiation dose was corrected by using an FWT nylon dosimeter of RCD radiometer film (FWT-60-00: Far West Technology, Inc., 330-D South Kellogg, Goleta, CA 93117, USA) with an irradiation reader (FWT-92D: Far West Technology, Inc., 330-D South Kellogg, Goleta, CA 93117, USA).

Based on mean density (*ρ*: kg/m^3^) and irradiation potential at specimen surface (*V*: keV), penetration depth (*D*th:/m) of HLEBI into the sample is expressed by Christenhusz and Reimer as [77]:

*D*th = 66.7*V* ^5/3^/*ρ* (1)

Since CF density is 1760 kgm^−3^ [66], *D*th is estimated to be 123 μm. If each CF ply thickness is assumed to be 286 μm after molding, there would be 286 − (123 + 123) = 40 μm at the core of each CF ply in which the HLEBI would not have penetrated. However, CF is highly conductive: CF, 6.5 μm in diameter and 10 mm in length, is reported to have an electrical conductivity of 6.67 × 10^4^ S/m [78]. For comparison, Al (annealed), Cu (annealed standard), and Fe (99.99+%) have conductivities on the order of 10^7^ S/m [79], while PC, being a strong insulator, has a conductivity of 10^−14^ S/m [5], and the CF diameter in this study is ~6 μm. Therefore, by applying HLEBI, electronic charge is assumed to activate throughout CF ply thickness. In addition, micro spaces between CFs in the cross-weave should also assist the HLEBI to penetrate further than the calculated *D*th. Namely, the HLEBI should activate the CF surface and generate DBs [80] to increase adhesion with PC.

Although the morphology data from the [PC]_4_[CF]_3_ samples to obtain exact thicknesses of each ply before and after HLEBI was not available at the time of this writing, HLEBI induces strength by generating nano-expansion sites in materials [68,81]. This is by repulsive forces at DB sites by lone pair electrons in close vicinity that create nano-compressive forces increasing the free volume [68,81]. From this, it is assumed HLEBI would slightly increase the thickness of each molded PC ply, and total [PC]_4_[CF]_3_ sample thickness by charge transfer from the CF.

### 2.3. Charpy Impact Test

To evaluate impact fracture toughness, Charpy impact values (*a*_uc_) of the [PC]_4_[CF]_3_ with and without HLEBI were measured using an impact fracture energy measurement system (Shimadzu Corporation No. 51735) conforming with JIS K 7077 standard [76] with the 80 × 10 × 2.0 mm samples. The impact value is expressed by the following equation [63].

*E* = *WR*[(cos*β* − cos*α*) − (cos*α*’ − cos*α*)(*α* + *β*)/(*α* − *α*’)] (2)

Here, *E*, *W*, *R*, *β*, *α*, and *α*’ are impact fracture energy (kJ), hammer mass (kg), length (m) of hammer weight point from rolling center, maximum angle after impact (Radians), start angle before impact (*α* = 2.3 Radians or 132°), and maximum angle of the blank test (Radians), respectively. The Charpy impact value (kJm^−2^) is expressed by the following equation:

*a*_uc_ = *E*/(*b* × *t*) (3)

Here, *E*, *b* (=10 ± 0.2 mm) and *t* (=2.00 ± 0.15 mm) are impact fracture energy (J), sample width (mm) and span distance (sample thickness, mm), respectively. The distance between supporting points was 40 mm.

### 2.4. Accumulative Probability

Evaluating the accumulative probability of strength at fracture (*P*_f_) is a convenient method of quantitatively analyzing experimental values, and in industry it is often employed in statistical quality control (QC). *P*_f_ is expressed by the following equation which is a generalized form of the median-rank method [82].

*P*_f_ = (*i* − 0.3)/(*N*_s_ + 0.4) (4)

Here, *N*_s_ and *i* are total number of samples and rank order integer of bending strength of each sample, respectively, where *i* is from weakest to strongest. In this case, *N*_s_ = 11 hence when *i* values are 1, 5, and 11, their corresponding *P*_f_ values are 0.07, 0.50 and 0.93, respectively.

### 2.5. X-Ray Photoelectron Spectroscopy of Untreated and HLEBI-Treated CF

A photoelectron spectrometer (XPS: Quantum 2000, ULVAC PHI Co., Chigasaki, Japan) was used to perform elemental analysis of CF untreated, and treated by 216 kGy, and 432 kGy HLEBI.

### 2.6. Calculation of Statistically Lowest Impact Value for Safety Design

For safety design, statistically lowest impact value, *a*_s_ at *P*_f_ = 0 is calculated for each data set using 3-parameter Weibull equation [83,84,85]:

*P*_f_ = 1 − exp [− ([*a*_uc_−*a*_s_]/*a*_III_) ^m^] (5)

Rearranged in linear form:

ln (− ln (1 − *P*_f_)) = *m* ln (*a*_uc_−*a*_s_) − *m* ln *a*_III_ (6)

When it is assumed the statistical Equation (2) is applicable to the experimental *a*_uc_ values, the *P*_f_ is dependent on risk of rupture [83,84,85]. The *a*_uc_, coefficient, *m* and constant (*a*_III_) are the important parameters in predicting strength requirements when screening new structural materials. The *a*_s_ at *P*_f_= 0 is obtained by iteration of Equation (6) until the correlation coefficient, *F*, reaches a maximum.

### 2.7. ANOVA Analysis for Significance of Differences Between [PC]_4_[CF]_3_ Data Sets

To validate the significance of observed differences in *a*_uc_ between the 6 data sets (Untreated, 43.2, 129, 216, 302, and 432 kGy) for the [PC]_4_[CF]_3_ composite, ANOVA single factor analysis was carried out since the HLEBI dose was the one variable in the experiment. Datapoints are the 11 Charpy impact values, *a*_uc_ (kJm^−2^) in each data set. Microsoft Excel was used with the “Data Analysis” Toolpack, where significance of threshold, α value was set at 0.01 indicating a 1% risk of erroneously concluding a difference between data sets exists. Parameters used are as follows. *F* is (MS_b_/MS_w_) ratio, where MS_b_ and MS_w_ are mean square values between (b), or within (w) data sets, respectively; *F_α_* is the threshold above which the Null Hypothesis does not hold; and *df* is number of degrees of freedom, equal to 5 in this case.

When the ANOVA program outputs *F* < *F_α_*, or *p*-value < *α* (0.01) the Null Hypothesis would not apply, i.e., one or more of the data sets is significantly different than the others. However, for the opposite output, (*F* > *F_α_*, or *p*-value > *α* (0.01)) ANOVA indicates the Null Hypothesis holds, signifying no significant difference exists between the data sets.

## 3. Results

### 3.1. Effect of CF Layer Addition on Impact Values of Untreated [PC]_4_[CF]_3_ Composite Compared to Untreated PC Resin

**Figure 4:**
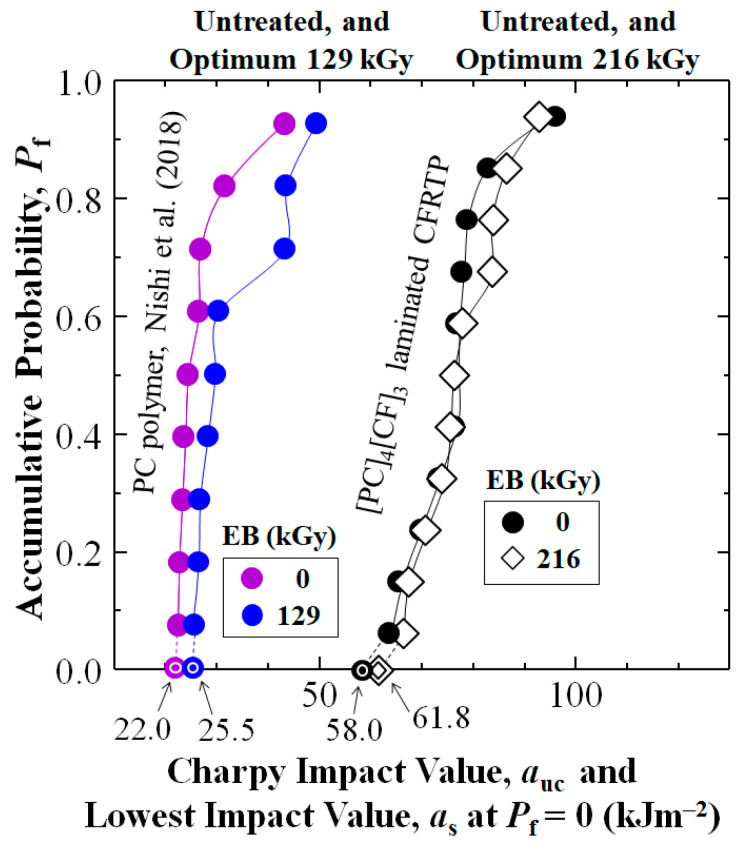
Accumulative probability, *P*_f_ vs. Charpy impact value, *a*_uc_, (kJm^−2^) for [PC]_4_[CF]_3_ laminated CFRTP along with data of PC resin from Nishi et al. (2018) [68]. Untreated and optimal HLEBI dose data sets are shown. Lowest statistical impact value, *a*_uc_ (*a*_s_ at *P*_f_ = 0) are indicated (arrows) for each data set.

Figure 4 first shows addition of cross-weave CF layer reinforcement to PC increased Charpy impact value, *a*_uc_ of the untreated [PC]_4_[CF]_3_ composite (black dots) over that of untreated PC resin (purple dots) reported by Nishi et al. (2018) [68] at all accumulative probabilities, *P*_f_. At low-, medial-, and high-*P*_f_ of 0.07, 0.50, and 0.93 *a*_uc_ was increased 2.81 (181%), 3.13 (213%), and 2.22 (122%) times from 22.6, 24.5, and 43.3 to 63.6, 76.6, and 96 kJm^−2^, respectively. The CF reinforcement also increased statistically lowest impact value *a*_s_ at *P*_f_ = 0, calculated by a 3-parameter Weibull equation (see Section 4.1) 2.64 times (164%) from 22.0 kJm^−2^ (purple bullseye) to 58.0 kJm^−2^ (black bullseye).

### 3.2. Effects of HLEBI Level on Impact Values of [PC]_4_[CF]_3_

**Figure 5:**
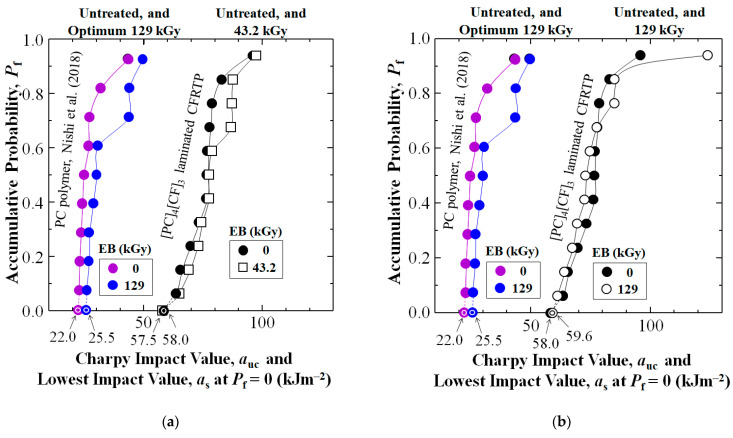

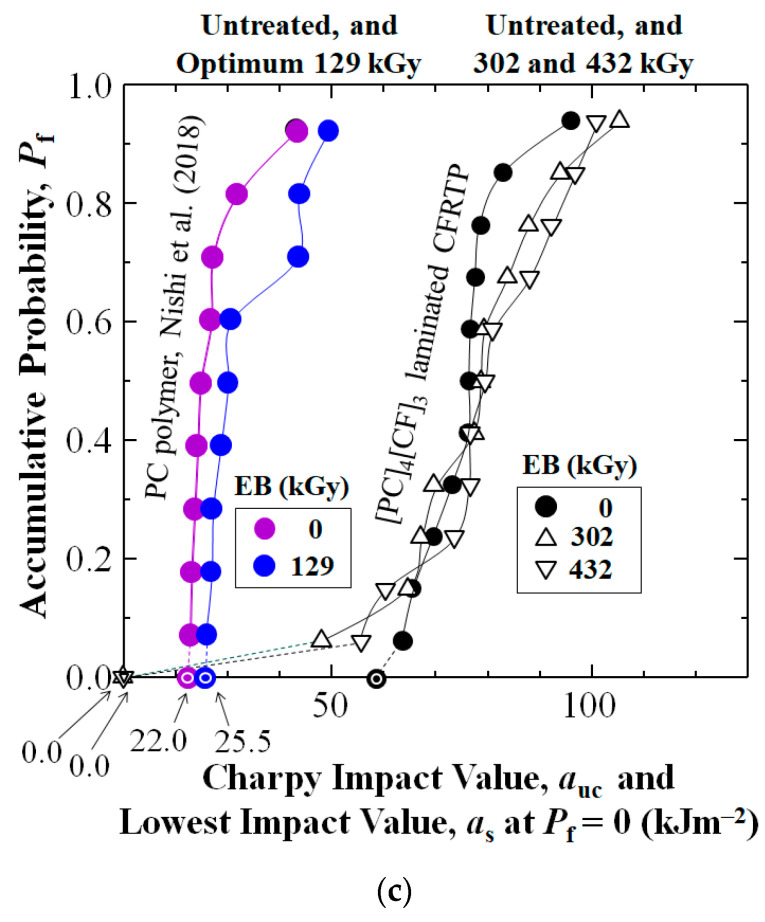
*P*_f_ vs. *a*_uc_ (kJm^−2^) data sets separated for clarity: (**a**) 43.2 kGy; (**b**) 129 kGy; and (**c**) 302 and 432 kGy HLEBI, along with untreated [PC]_4_[CF]_3_. Again, the data for PC resin and PC resin by optimal 129 kGy HLEBI from Nishi et al. (2018) [68] is shown for comparison. Lowest statistical impact value, *a*_uc_ (*a*_s_ at *P*_f_ = 0) are indicated (arrows) for each data set.

Figure 4 also shows the *a*_uc_ data sets for optimum HLEBI doses to [PC]_4_[CF]_3_ composite, and that reported for PC resin (blue dots) [68], respectively. For the [PC]_4_[CF]_3_ applying 216 kGy raised *a*_s_ at *P*_f_ = 0 about 6.5% from 58.0 kJm^−2^ for untreated (black dots) to 61.8 kJm^−2^ (diamonds). Interestingly for PC resin, the reported optimum HLEBI dose was less, at 129 kGy, resulting in 16% increase in *a*_s_ at *P*_f_ = 0, from 22.0 to 25.5 kJm^−2^ [68]. For the [PC]_4_[CF]_3_, Table 1 along with Figure 4 and Figure 5 show the 216 kGy HLEBI exhibited the highest *a*_s_ at *P*_f_ = 0 at 61.8 kJm^−2^, along with the lowest standard deviation (8.30 kJm^−2^), hence, 216 kGy is considered the optimum for [PC]_4_[CF]_3_.

Since the remaining data sets of untreated, 43.2, 129, 302, and 432 kGy HLEBI to [PC]_4_[CF]_3_ had overlapping values with that of 216 kGy, Figure 5a–c show them separately for clarity. PC data sets in [68] are included for easy comparison. Figure 5a shows the 43.2 kGy HLEBI dose slightly increases *a*_uc_ at all *P*_f_, but *a*_s_ at *P*_f_ = 0 is decreased slightly. Figure 5b shows 129 kGy lowers *a*_uc_ at most *P*_f_ except for significant increase at highest *P*_f_ of 0.93. Figure 5c shows higher 302 and 432 kGy doses resulted in higher *a*_uc_ at *P*_f_ > 0.40, but the *a*_s_ at *P*_f_ = 0 was dropped to 0 kJm^−2^. Similarly, Table 1 shows some of the *a*_uc_ values at median-*P*_f_ = 0.50 are higher than those of the 216 kGy, but the 216 kGy samples give the slightly higher *a*_s_ at *P*_f_ = 0.

Note, it would be valuable to have conducted batch studies to increase reliability of tests. Usually, to test against bird strike or other damage, batches of specimens from several different vendors are tested before qualification of a material for maximum safety. For aircraft CFRPs, Charpy impact tests are utilized to provide a preliminary estimation on which materials should be chosen for further tersting such as compression after impact, or drop tower test. However, available samples were limited, hence batch studies were beyond the scope of this study.

The 3-parameter Weibull analysis calculated by plots as in Figure 4 has been a widely used method in industrial quality control (QC) to calculate the lowest statistically impact value *a*_s_ at *P*_f_ = 0 to assess lower limit of safety of parts [83,84,85]. The data in Figure 4 clearly shows that the [PC]_4_[CF]_3_ composite samples gave significantly higher *a*_uc_ values than that of PC resin. Also, for the [PC]_4_[CF]_3_ samples, 3-parameter Weibull analysis, resulting in the 6.5% increase in the 216 kGy over that of untreated CF is considered significant [83,84,85].

## 4. Discussion

### 4.1. Estimation of Lowest Statistical Impact Value, a_s_ at P_f_ = 0 for Safety

**Figure 6:**
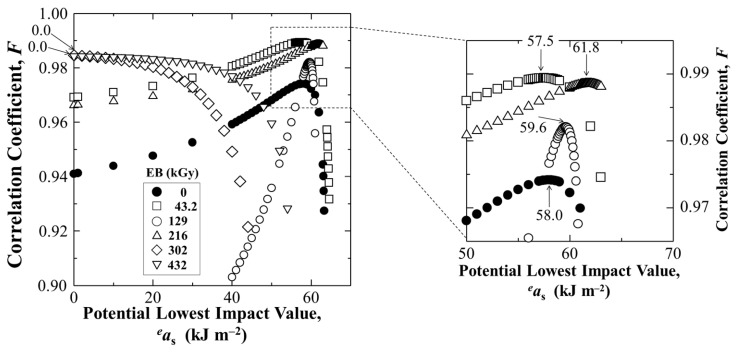
Changes in correlation coefficient (*F*) versus the potential *a_s_* value (*^e^a_s_*) for untreated and HLEBI irradiated [PC]_4_[CF]_3_ samples. The lowest impact value, *a_s_* (arrows) is determined at maximum *F*.

**Figure 7:**
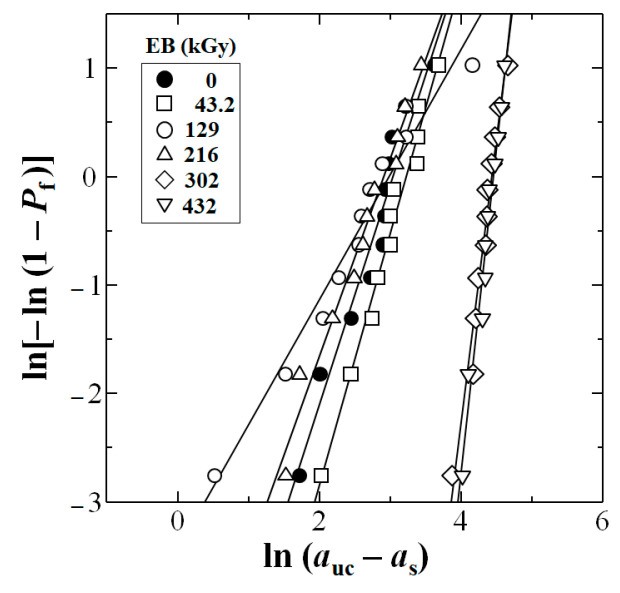
Linear relationships between ln(*a*_uc_ − *a*_s_) and ln[−ln(1 − *P*_f_)] from Weibull 3-D calculation for untreated and HLEBI irradiated [PC]_4_[CF]_3_ samples, respectively.

Figure 6 shows estimation of lowest statistical impact value, *a*_s_ at *P*_f_ = 0 Weibull 3-D calculation for untreated and HLEBI irradiated [PC]_4_[CF]_3_ samples, respectively, using Equation (2). The HLEBI of 216 kGy yields the highest *a*_s_ at *P*_f_ = 0 of 61.8 kJm^−2^. Figure 7 exhibits the obtained linear relationships between ln(*a*_uc_ − *a*_s_) and ln[−ln(1 − *P*_f_)].

### 4.2. ANOVA Analysis

Table 2 shows ANOVA analysis of the data sets. The *p*-value (0.98589) is much higher than *α* (0.01), and *F_α_* (3.33888) > *F* (0.12637), indicating the Null Hypothesis holds, signifying no significant difference exists between data sets. However, in engineering of new materials, Weibull 3-parameter analysis is often used in quality control (QC) to assess the safety of parts and clearly takes into account if the weakest sample(s) in the data set are much weaker than the others [83,84,85]. The statistically lowest strength, *a*_s_ at *P*_f_ = 0 is a key parameter in designing new materials to pass the requirements for further testing to obtain maximum safety [83,84,85].

### 4.3. Charpy Impact Value vs. HLEBI Dose as a Function of Accumulative Probability and Lowest Statistical Value for Safety

**Figure 8:**
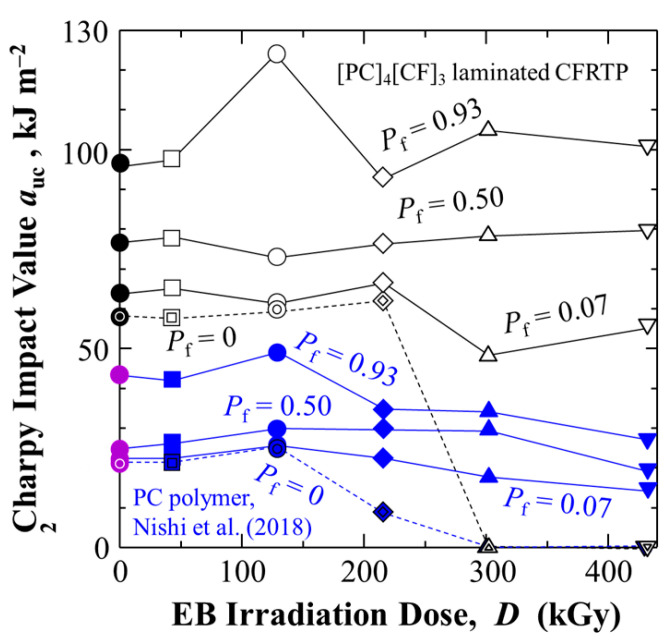
Charpy impact value, *a*_uc_ (kJm^−2^) vs. HLEBI dose, *D* (kGy) at low- (0.07), media-l (0.50), and high- (0.93) *P*_f_ (solid lines) for the [PC]_4_[CF]_3_ samples. Those of PC polymer samples in Nishi et al. (2018) [68] are also shown (untreated in purple, HLEBI treated in blue). Statistically lowest *a*_uc_ (*a*_s_) at *P*_f_ = 0 are included (dotted lines).

Figure 8 shows plots of *a*_uc_ vs. HLEBI dose at low- (0.07), medial- (0.50), and high-(0.93) *P*_f_, along with *a*_s_ at *P*_f_ = 0. Regardless of HLEBI dose, addition of CF in [PC]_4_[CF]_3_ (black lines) substantially raises the *a*_uc_ over those reported for PC resin (blue lines) [68]. Here, optimum 216 kGy dose data are shown with increased *a*_s_ at *P*_f_ = 0 from 58.0 to 61.8 kJm^−2^. Interestingly, the *a*_s_ at *P*_f_ = 0 maintains stable values followed by dropping off as HLEBI dose is increased. The dropoff for PC resin begins above optimum 129 kGy, while that for [PC]_4_[CF]_3_ starts above optimum 216 kGy. This enhancement is most likely due to halting of cracks in the PC by the CF. Purple data points are those reported for PC untreated.

### 4.4. HLEBI-Induced Adhesion Enhancement at PC/CF Interface: ESR and XPS

**Figure 9:**
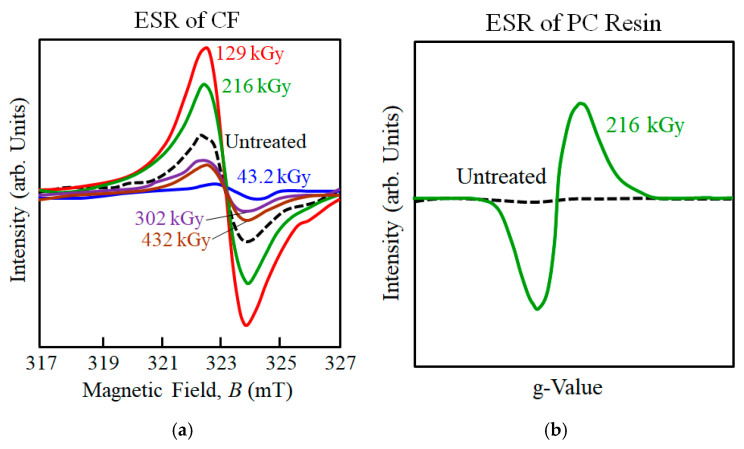
(**a**,**b**). ESR signals of (**a**) CF untreated and directly treated with 43.2 to 432 kGy-HLEBI; and (**b**) PC untreated and directly treated with 216 kGy-HLEBI. (**a**) is adapted from Nishi et al. (2023) [66]; (**b**) is adapted from Nishi et al. (2005) [86].

To discuss how 216 kGy HLEBI modifies the surface chemistry of CF and propose a mechanism of how bonding is enhanced with PC, electron spin resonance (ESR) and X-ray photoelectron spectroscopy (XPS) results are covered here. Table 3 shows optimal HLEBI dose for impact is congruent with experimental results for 3-point bending, both at 216 kGy, while Figure 9a,b show 216 kGy generates significant DBs in both CF [66] and PC [86], as evidenced by strong ESR peaks.

Figure 9a shows when HLEBI is applied directly to CF, with increasing dose the ESR peak height decreases, then increases, then decreases [66]. ESR detects magnetic fields by lone pair electrons, i.e., DBs. Untreated CF emits a peak due to naturally occurring DBs likely due to imperfections in the graphite hexagonal structure [66]. However, at 43.2 kGy dose, ESR peak height is decreased indicating decrease in DB density; which can be explained by HLEBI forcibly diffusing carbon atoms to fill vacant sites and disjointed interfaces between hexagonal graphitic planes. However, at the higher 129 and 216 kGy, strong ESR peak heights are generated indicating substantial increase in BD density [66], for strong bonding with PC. However, higher doses of 302 and 432 kGy show decrease in peak intensity [66], which can be explained by the excess dose forming interstitial atoms between graphitic planes increasing entropy, and decreasing the regularity of the graphitic crystal structure. When electrons are transferred to the PC, Figure 9b shows 216 kGy HLEBI will generate DBs in the PC.

**Figure 10:**
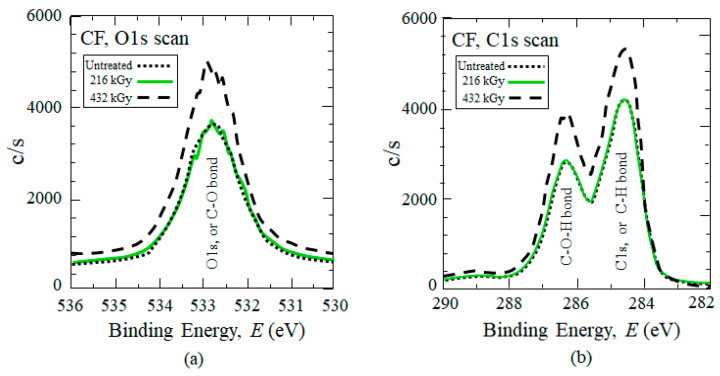
(**a**,**b**) XPS signals on CF untreated and directly treated with 216 and 432 kGy-HLEBI.

To discuss the surface chemistry, Figure 10a,b show XPS scans of CF, while Table 4 lists chemical groups on CF and PC [69] surfaces. Oxygen atoms typically exist at the CF surface [87]. XPS analyzes surface chemistry to a depth of 5 to 10 nm [88], and sizing layer on commercial CFs is reported as generally thicker, about 100 nm (0.1 μm) [89,90]. Thus, the appearance of O groups would apparently be from: (1) the CF sizing, which is typically an epoxy that contains O; (2) residual < 300 ppm O_2_ in the HLEBI chamber; and (3) O groups existing on bare CF itself [87], at sites possibly cleaned by the HLEBI. For untreated CF, Figure 10a shows XPS O1s scan results in a single peak at 532.8 eV indicating presence of C–O bonds, while Figure 10b shows C1s scan generating two peaks at about 286.3 and 284.4 eV, representing C–O–H and C– H bonds, respectively.

For the 216 kGy CF sample, although it appears to be optimum for *a*_uc_ of [PC]_4_[CF]_3_, no changes in XPS peak heights are observed, indicating little or no effect on C–O–H, C–H, or C-O bonds. However, at 432 kGy the XPS data in Figure 10a,b show increases the O-H, C–H, or C–O bonds, which would act to increase PC/CF interfacial adhesion in the [PC]_4_[CF]_3_ composite in the form of CF:C:O:C:PP and CF:C:C:PP bonds preventing CF pullout.

As mentioned earlier, electron charge will transfer from the CF plies to the PC during laminate assembly and hot press. Table 4 includes XPS results by Nathawat et al. (2007) who found chemical groups detected on PC film surface were C–H, C–C, C–O–H, C–O–C, and O=C=O by C1s scan, and C=O, O–C=O, and C–O–C=O by O1s scan [69]. The film was treated with 280 kGy electron beam, resulting in O concentration increase from 5.75% to 8.0% [69].

### 4.5. Mechanism of Nano-Strengthening by HLEBI-Induced Adhesion Enhancement at PC/CF Interface

**Figure 11:**
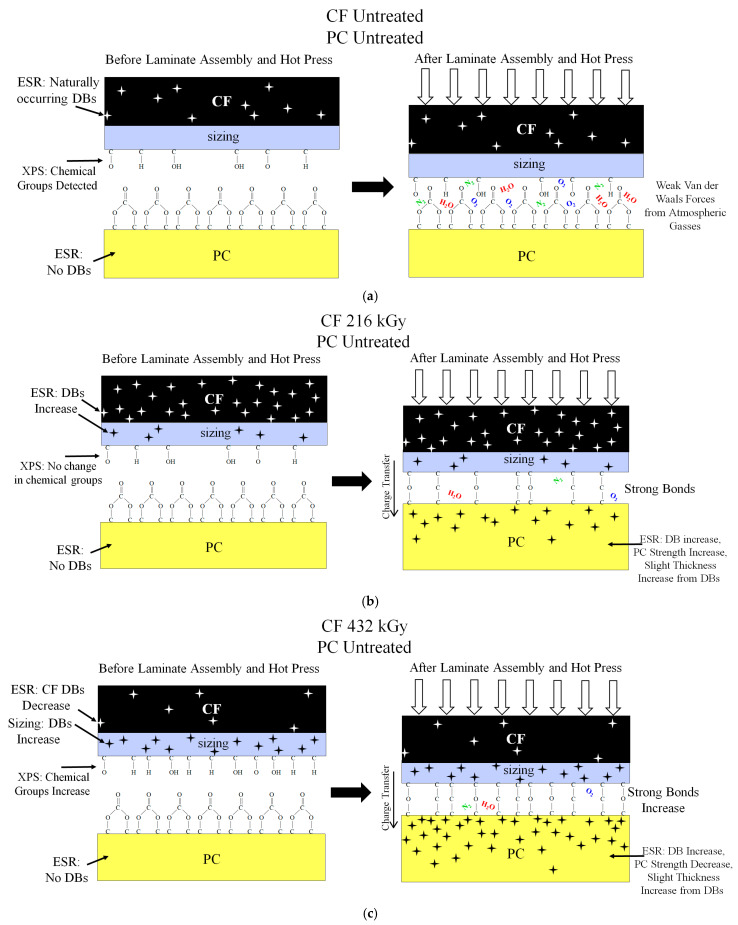
Illustration of surface chemistry of PC [69,91] and CF interface: (**a**) untreated, (**b**) treated with 129 kGy, and (**c**) 432 kGy HLEBI. Stars indicate dangling bonds. Not drawn to scale. Only one interface is shown for clarity.

To discuss the nano-strengthening mechanism of the PC/CF interface by HLEBI to CF in [PC]_4_[CF]_3_, Table 4 lists bonds detected on CF and PC surface [69] detected by XPS, while Figure 11a–c illustrates the action for CF untreated, 216 kGy, and 432 kGy samples. For untreated CF, Figure 11a shows **before laminate assembly and hot press**, untreated CF has naturally occurring DBs (stars) detected by ESR [66]; with chemical groups C-O and C-OH detected by XPS (Figure 10); and as mentioned above, untreated PC has no DBs found by ESR; and C–H, C–C, C–O–H, C–O–C, O=C=O, C=O, O–C=O, and C–O–C=O groups detected by XPS [69]. **After laminate assembly and hot press**, Figure 11a shows the untreated sample has weak Van der Waals adhesive forces from atmospheric gasses N_2_, O_2_, and H_2_O resulting in poor adhesion [66].

For the 216 kGy samples, Figure 11b shows **before laminate assembly and hot press**, DBs are increased significantly in CF by 216 kGy HLEBI as detected by ESR and are assumed to exist in the CF sizing as well. However, XPS data in Figure 10a,b show no change in number of chemical groups, i.e., no change in XPS peak heights, compared to untreated. This shows 216 kGy HLEBI did not increase chemical groups on the CF surface. However, **after laminate assembly and hot press**, in the 216 kGy sample strong bonds at the PC/CF interface are created as evidenced by rise in *a*_s_ at Pf = 0. This is most likely due to charge transfer to the PC leading to DB generation in PC. Slight increases in PC thickness should occur from free volume increase by DBs [68,81]. It is reported 216 kGy to PC resin increases *a*_uc_ while 129 kGy is optimum [86].

For the 432 kGy samples, Figure 11c shows **before laminate assembly and hot press**, DBs are decreased significantly in CF to less than that of untreated as detected by reduced ESR peak in Figure 9a. However, XPS data in Figure 10a,b show an increase in number of chemical groups, i.e., increased XPS peak heights, compared to untreated and 216 kGy. This may be due to higher crystal perfection in the graphite structure by the HLEBI with DBs reduced, but more charge to the cleaned CF surface or sizing. **After laminate assembly and hot press**, in the 432 kGy sample increase in strong bonds at the PC/CF interface are generated as evidenced by rise in *P*_f_ between 0.50 and 0.93. However, as mentioned earlier Figure 5c shows *a*_uc_ was reduced in the two weakest samples in the data set at *P*_f_ of 0.06 and 0.15, and *a*_s_ at *P*_f_ = 0 was reduced to 0 kJm^−2^ indicating insufficient quality and safety. Although the high 432 kGy dose increased strong bonds at the PC/CF interface acting to increase *a*_uc_ at the higher *P*_f_, the tradeoff was apparently excess DBs transferring to the PC, weakening it. This is supported by the literature that 432 kGy HLEBI to PC resin significantly reduces *a*_uc_ at all *P*_f_ as damage from excess DBs [86]. Thus, for [PC]_4_[CF]_3_, 216 kGy HLEBI to CF appears to be a well-balanced condition between PC-cohesive force and PC/CF interface adhesive force.

### 4.6. Difference in Action of HLEBI-Generated Strong Bonds with Test Type

**Figure 12:**
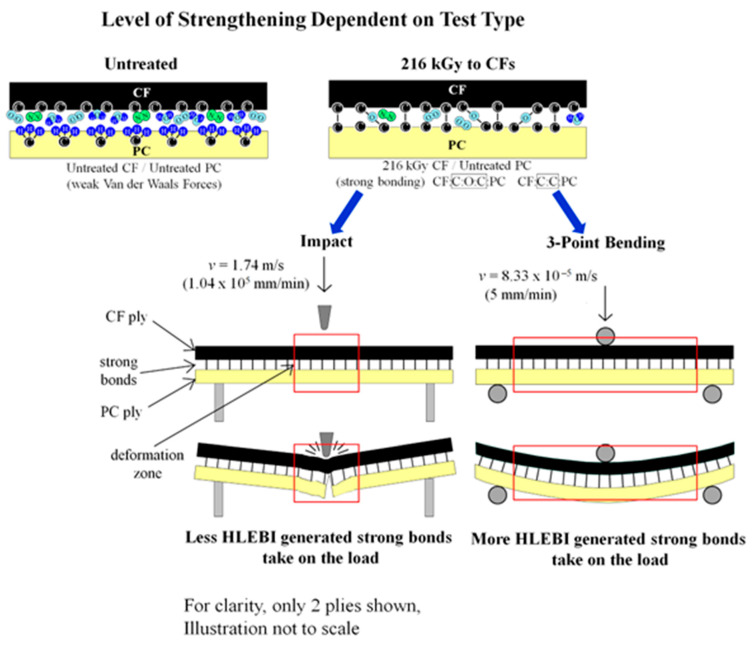
Mechanism of improvement difference between impact, and that reported for 3-point bending of the [PC]_4_[CF]_3_ composite from Nishi et al. (2018) [66] treated by optimal 216 kGy HLEBI.

As mentioned earlier, the 216 kGy as optimum for impact strength for [PC]_4_[CF]_3_ agrees with a previous study by Nishi et al. (2023) [66] that showed 216 kGy to be optimum for 3-point bending strength, when quality can be controlled. However, bending strength parameters were improved much more over untreated than those of impact. Table 4 shows impact parameters, *a*_s_ at *P*_f_ = 0 and *a*_uc(avg)_ were improved by 6.5% and 2.1%, respectively, while bending strength parameters, *σ*_s_ at *P*_f_ = 0 and *σ*_s(avg)_ were improved by 18.6% and 19.8% [66]. Figure 12 illustrates that the mechanism of improvement difference between impact and bending is apparently dependent on deformation zone and speed. First, for untreated [PC]_4_[CF]_3_ samples, atmospheric gases such as N_2_, O_2_, and H_2_O exist at the PC/CF interface create weak Van der Waals attractive forces. When 216 kGy HLEBI is applied to the CFs, DBs are generated and charge transfer occurs to the PC, generating strong of CF:C:O:C:PC and CF:C:C:PC bonds enhancing adhesion around CF circumference preventing fiber pullout. Assuming bond density generation is constant since HLEBI dose is constant, the strength increase should be dependent on deformation area and velocity. The impact test pendulum is at high velocity, *v* of 1.74 m/s (1.04 × 10^5^ mm/min) [92] at point of impact, hence deformation zone is smaller (red box), with less HLEBI generated bonds to take on the load. On the other hand, the bending test is at lower *v* of 8.33 × 10^−5^ m/s (5 mm/min) [66] with larger deformation zone (red box), thus more HLEBI generated bonds take on the load. As illustrated in Figure 12, impact involves a significantly smaller deformation area than bending, with the small hemispheric tip of the impactor hitting rapidly, as opposed to the slower deformation of bending forming the specimen gradually into an arc before fracture. In summary, for HLEBI improvements, the level of strengthening appears to be dependent on mechanical test type for the [PC]_4_[CF]_3_ composite.

### 4.7. Benefits, Potential Practical Applications, and Future Research

In addition to those mentioned in the introduction, benefits of this research include [PC]_4_[CF]_3_ can be treated with HLEBI internally throughout the bulk for any panel thickness prior to laminate assembly and hot press. Of course, recyclability of PC and CF is the largest benefit to lower carbon footprint and save on petroleum extraction and material costs. As mentioned earlier, the production cycle of TP is about 1/10th that of epoxies to conserve energy and costs. Importantly, PC itself is easily recyclable, therefore recycled PC can be used for CFRPC fabrication lowering material cost. Although melting PC out of CFTPC to recover the PC and CF is still in the development phase, it is possible the CFRPC composite can be re-melted or shredded to be useful as scrap to be reformed, but more research is needed. Potential practical applications for CFRPC layered composites with stronger impact strength include industries of aerospace (A380 Airbus leading edge of main wings, electronics casings), space vehicles (electronics casings, propeller for Mars helicopter, launch and re-entry vehicles), automotive (car chassis, covers for engine compartments, interior parts), sports equipment (snowboards), electronics (home appliances, electronics, PC, iPhone covers), and building construction. For maximum safety, it is strictly recommended to adjust for optimum HLEBI dose for each part configuration. Details of future research are proprietary but should include batch studies to use for scale-up for practical articles, and aging studies to assess any loss in strength with time.

## 5. Conclusions

With the increase in extreme weather events and depletion of natural resources, it is vital to transition to a circular economy. Polycarbonate (PC) has the environmental benefit of being a recyclable thermoplastic (TP) that can be repeatedly melted and solidified to overcome single use plastic, landfill waste, and incineration for a cleaner and more sustainable environment. However, there exists little research on electron beam to increase mechanical properties of carbon fiber reinforced polycarbonate (CFRPC). The goals of this research were two-fold. First, we demonstrated high impact strength of PC resin can be raised 2 to 3 times by adding interlayered carbon fiber (CF) plies between PC sheets by hot press. Second, we showed for the resulting CFRTP, impact strength of the weakest samples in a data set can be increased further by using homogeneous low voltage electron beam irradiation (HLEBI) without the use of additives, chemicals, or particles. HLEBI does this by generating dangling bonds (DBs) resulting in strong bonds at the PC/CF interface, and nano-compressive forces within materials when optimum dose is determined. Mainly, the weakest samples in the data set are strengthened indicating an increase in safety and the reliability of parts. This is with the ultimate goal of strengthening CFRTPs to replace those of non-recyclable epoxy CFRPs for aerospace, automotive, building construction, and sports equipment with highest concern for safety and a sustainable environment.

## Figures and Tables

**Table 1:** Average *a*_uc_, *a*_uc_ at median-*P*_f_ = 0.5, and *a*_s_ at *P*_f_ = 0 (kJm^−2^) for HLEBI doses and untreated for [PC]_4_[CF]_3_ along with data of PC resin in [68].

	PC	PC	[PC]_4_[CF]_3_	[PC]_4_[CF]_3_	[PC]_4_[CF]_3_	[PC]_4_[CF]_3_	[PC]_4_[CF]_3_	[PC]_4_[CF]_3_
HLEBI dose (kGy)	Untreated	129	Untreated	43.2	129	216	302	432
*a*_uc,(avg)_ (kJm^−2^) (Std. Dev.)	27.2 (6.65)	33.8 (9.09)	76.1 (8.79)	79.6 (9.45)	78.6 (19.9)	77.7 (8.30)	77.8 (15.5)	80.1 (14.1)
*a*_uc_ at *P*_f_ = 0.50 (kJm^−2^)	24.5	28.4	76.6	77.7	73.0	76.3	78.6	79.4
*a*_s_ at *P*_f_ = 0 (kJm^−2^)	22.0	25.5	58.0	57.5	59.6	61.8	0	0

**Table 2:** ANOVA analysis results of the *a*_uc_ data sets with *α* set at 0.01.

	SS	df	MS	F	*p*-Value	*F*_crit_ (*F_α_*)
Variance Between Groups	112	5	22.5	0.12637	0.98589	3.33888
Variance Within Groups	10,682	60	178.0			
TOTAL	10,794	65				

**Table 3:** Differences in improvements between impact, and that reported for 3-point bending of the [PC]_4_[CF]_3_ composite [66] treated by optimal 216 kGy HLEBI.

	Impact (kJm^−2^)	3-Point Bending (MPa)
*a*_s_, or *s*_s_ at *P*_f_ = 0	6.5%	18.6%
*a*_uc(avg)_, or *s*_b(avg)_	2.1%	19.8%

**Table 4:** Chemical groups at surfaces of CF and PC [69] by XPS analysis.

Scan	CF	PC
O 1s	C–O	C=O; O–C=O; C–O–C=O
C 1s	C–O–H; C–H	C–H, C–C, C–O–H, C–O–C

## Data Availability

Data are available upon request from Michael Faudree of Tokyo City University (faudree@tcu.ac.jp), and Yoshitake Nishi from Tokai University (west@tsc.u-tokai.ac.jp).

## References

[1] Plastic Pollution is Growing Relentlessly as Waste Management and Recycling Fall Short, Says OECD. Organisation for Economic Co-operation and Development Press Release 2/22/2022. https://www.oecd.org/en/about/news/press-releases/2022/02/plastic-pollution-is-growing-relentlessly-as-waste-management-and-recycling-fall-short.html.

[2] Nomura R., Kanda M., Faudree M.C., Jimbo I., Nishi Y. (2014). Improving impact value of interlayered glass fiber chopped strand mat reinforced thermoplastic polypropylene externally irradiated by homogeneous low potential electron beam. Mater. Trans..

[3] Nomura R., Kanda M., Faudree M.C., Jimbo I., Nishi Y. (2016). Internal activation of thermoplastic polypropylene GFRTP composite by homogeneous low energy electron beam irradiation (HLEBI) of the interlayered glass fiber chopped strand mats (GF-CSM) prior to assembly. Mater. Trans..

[4] Polycarbonate (PC): Understand the Key Benefits and Applications. https://www.protolabs.com/materials/polycarbonate/.

[5] James A., Lord M. (1992). Macmillan’s Chemical and Physical Data, London and Basingstoke.

[6] Gordon A., Ford R. (1972). The Chemist’s Companion: A Handbook of Practical Data, Techniques, and References.

[7] Tuteja B., Khan F., Sundararajan P. (2005). Re-Plasticization by Confinement During Annealing Induced Phase Separation in Polycarbonate/Phthalate Plasticized Films. Macromol. Chem. Phys..

[8] Ueda H., Okumura W., Uematsu H., Tanoue S. (2016). Processing of Carbon Fiber Fabric Reinforced Polycarbonate–Formation Mechanism of Void. J. Fiber Sci. Technol..

[9] Kitagawa S., Kimura H., Uchida H.T., Faudree M.C., Tonegawa A., Kaneko S., Salvia M., Nishi Y. (2019). A new process of thermoplastic polypropylene reinforced by interlayered activated carbon fiber treated by electron beam irradiation under nitrogen gas atmosphere with oxygen prior to assembly and hot-press. Mater. Trans..

[10] Dilsiz N., Wightman J.P. (2000). Effect of acid-base properties of unsized and sized carbon fibers on fiber/epoxy matrix adhesion. Colloids. Surf. A.

[11] Allred R.E., Wesson S.P., Shin E.E., Inghram L., McCorkle L., Papadopoulos D., Wheeler D., Sutter J.K. (2003). The influence of sizings on the durability of high-temperature polymer composites. High Perform. Polym..

[12] Drzal L.T., Raghavendran V.K. (2003). Adhesion of thermoplastic matrices to carbon fibers: Effect of polymer molecular weight and fiber surface chemistry. J. Thermoplast. Compos. Mater..

[13] Kettle A.P., Beck A.J., O’Toole L., Jones F.R., Short R.D. (1997). Plasma polymerisation for molecular engineering of carbon fibre surfaces for optimised composites. Compos. Sci. Technol..

[14] Zhang C.H., Zhang Z.Q., Cao H.L. (2007). Effects of epoxy/SiO_2_ hybrid sizing on the mechanical properties of carbon fiber composites. Solid State Phenom..

[15] Tiwari S., Bijwe J. (2014). Surface treatment of carbon fibers: A review. Procedia Tech..

[16] Sharma M., Gao S., Mader E., Sharma H., Wei L.-Y., Bijwe J. (2014). Carbon fiber surfaces and composite interphases. Compos. Sci. Technol..

[17] Xu Z., Chen L., Huang Y., Li J., Wu X., Li X., Jio Y. (2008). Wettability of carbon fibers modified by acrylic acid and interface properties of carbon fiber/epoxy. Eur. Polym. J..

[18] Wu Z., Pittman C.U., Gardner S.D. (1995). Nitric acid oxidation of carbon fibers and the effects of subsequent treatment in refluxing aqueous NaOH. Carbon.

[19] Cai G., Wada M., Ohsawa I., Kitaoka S., Takahashi J. (2019). Interfacial adhesion of recycled carbon fibers to polypropylene resin: Effect of superheated steam on the surface chemical state of carbon fiber. Compos. A.

[20] Tiwari S., Sharma M., Panier S., Mutel B., Mitschang P., Bijwe P. (2011). Influence of cold remote nitrogen oxygen plasma treatment on carbon fabric and its composites with specialty polymers. J. Mater. Sci..

[21] Pittman C.U., Jiang W., He G.R., Gardner S.D. (1998). Oxygen plasma and isobutylene plasma treatments of carbon fibers: Determination of surface functionality and effects on composite properties. Carbon.

[22] Sherwood P.M.A. (1996). Surface analysis of carbon and carbon fibers for composites. J. Electron Spectrosc. Relat. Phenom..

[23] Montes-Morán M.A., Martínez-Alonso A., Tascón J.M.D., Young R.J. (2001). Effects of plasma oxidation on the surface and interfacial properties of ultra-high modulus carbon fibres. Compos. Part A.

[24] Hung B., Li J., Fan Q., Chen Z.H. (2008). The enhancement of carbon fiber modified with electropolymer coating to the mechanical properties of epoxy resin composites. Compos. Part A.

[25] Bao D., Cheng X. (2006). Evaluation of tribological performance of PTFE composite filled with rare earths treated carbon fibers under water-lubricated condition. J. Rare Earths.

[26] Zhang X.R., Zhao P., Pei X.Q., Wang Q.H., Jia Q. (2007). Flexural strength and tribological properties of rare earth treated short carbon fiber/polyimide composites. Express Polym. Lett..

[27] Clough R.L. (2001). High-energy radiation and polymers: A review of commercial processes and emerging applications. Nucl. Instrum. Methods Phys. Res. Sect. B.

[28] Xu Z., Huang Y., Zhang C., Liu L., Zhang Y., Wang L. (2007). Effect of γ-ray irradiation grafting on the carbon fibers and interfacial adhesion of epoxy composites. Compos. Sci. Technol..

[29] Wan Y.Z., Wang Y.L., Huang Y., Luo H.L., Chen G.C., Yuan C.D. (2005). Effect of surface treatment of carbon fibers with gamma-ray radiation on mechanical performance of their composites. J. Mater. Sci..

[30] Li J., Huang Y., Xu Z., Wang Z. (2005). High-energy radiation technique treat on the surface of carbon fiber. Mater. Chem. Phys..

[31] Guo J., Lu C., An F., He S. (2012). Preparation and characterization of carbon nanotubes/carbon fiber hybrid material by ultrasonically assisted electrophoretic deposition. Mater. Lett..

[32] Lee S.B., Choi O., Lee W., Yi J.W., Kim B.S., Byun J.H., Yoon M.K., Fong H., Thostenson E.T., Chou T.W. (2011). Processing and characterization of multi-scale hybrid composites reinforced with nanoscale carbon reinforcements and carbon fibers. Compos. Part A.

[33] Li J. (2008). Interfacial studies on the O_3_ modified carbon fiber-reinforced polyamide 6 composites. Appl. Surf. Sci..

[34] Liu L., Song Y.J., Fu H.J., Jiang Z.X., Zhang X.Z., Wu L.N., Huang Y.D. (2008). The effect of interphase modification on carbon fiber/polyarylacetylene resin composites. Appl. Surf. Sci..

[35] Fukunaga A., Ueda S., Nagumo M. (1999). Air-oxidation and anodization of pitch-based carbon fibers. Carbon.

[36] Fukunaga A., Ueda S. (2000). Anodic surface oxidation for pitch-based carbon fibers and the interfacial bond strengths in epoxy matrices. Compos. Sci. Technol..

[37] Park S.J., Kim M.H. (2000). Effect of acidic anode treatment on carbon fibers for increasing fiber–matrix adhesion and its relationship to interlaminar shear strength of composites. J. Mater. Sci..

[38] Guo H., Huang Y.D., Meng L.H., Liu L., Fan D.P., Liu D.X. (2009). Interface property of carbon fibers/epoxy resin composite improved by hydrogen peroxide in supercritical water. Mater. Lett..

[39] Severini F., Formaro L., Pegoraro M., Posca L. (2002). Chemical modification of carbon fiber surfaces. Carbon.

[40] Tiwari S., Bijwe J., Panier S. (2011). Tribological studies on polyetherimide composites based on carbon fabric with optimized oxidation treatment. Wear.

[41] Joo J.-H., Kim S.-H., Yim Y.-J., Bae J.-S., Seo M.-K. (2025). Interfacial Interlocking of Carbon Fiber-Reinforced Polymer Composites: A Short Review. Polymers.

[42] Park S.J., Seo M.K., Rhee K.Y. (2003). Studies on mechanical interfacial properties of oxyfluorinated carbon fibers-reinforced composites. Mater. Sci. Eng. A.

[43] Nishi Y., Tsuchikura N., Nanba S., Yamamoto T., Faudree M.C. (2012). Charpy Impact of Sandwich Structural Composites (CFRP/PC/CFRP) of Polycarbonate (PC) Cores Covered with Carbon Fiber Cross Textile Reinforced Epoxy Polymer (CFRP) Thin Sheets as a Function of Temperature. Mater. Trans..

[44] Jahangir M.N., Billah K.M.M., Lin Y., Roberson D.A., Wicker R.B., Espalin D. (2019). Reinforcement of material extrusion 3D printed polycarbonate using continuous carbon fiber. Addit. Manuf..

[45] Yao T.-T., Wu G.-P., Song C. (2017). Interfacial adhesion properties of carbon fiber/polycarbonate composites by using a single-filament fragmentation test. Compos. Sci. Technol..

[46] Ozkan C., Gamze Karsli N., Aytac A., Deniz V. (2014). Short carbon fiber reinforced polycarbonate composites: Effects of different sizing materials. Compos. B Eng..

[47] Hacioglu F., Tayfun U., Ozdemir T., Tincer T. (2021). Characterization of carbon fiber and glass fiber reinforced polycarbonate composites and their behavior under gamma irradiation. Prog. Nucl. Energy.

[48] Yao T.-T., Liu Y.-T., Zhu H., Zhang X.-F., Wu G.-P. (2019). Controlling of resin impregnation and interfacial adhesion in carbon fiber/polycarbonate composites by a spray-coating of polymer on carbon fibers. Compos. Sci Technol..

[49] Yao T.-T., Zhang X.-F., Zhang W.-S., Liu Y.T., Liu Q., Wu G.-P. (2021). Controlled attachment of polycarbonate nanoparticles on carbon fibers for increased resin impregnation and interfacial adhesion in carbon fiber composites. Compos. Part B Eng..

[50] Kamps J.H., Scheffler C., Simon F., van der Heijden R., Verghese N. (2018). Functional polycarbonates for improved adhesion to carbon fibre. Compos. Sci. Technol..

[51] Wu Y., Dhamodharan D., Wang Z., Wang R., Wu L. (2020). Effect of electrophoretic deposition followed by solution pre-impregnated surface modified carbon fiber-carbon nanotubes on the mechanical properties of carbon fiber reinforced polycarbonate composites. Compos. Part B Eng..

[52] Baek Y.-M., Shin P.-S., Kim J.-H., Park H.-S., Lawrence DeVries K., Park J.-M. (2020). Thermal transfer, interfacial, and mechanical properties of carbon fiber/polycarbonate-CNT composites using infrared thermography. Polym. Test..

[53] Cho B.G., Hwang S.-H., Park M., Park J.-K., Park Y.-B., Chae H.-G. (2019). The effects of plasma surface treatment on the mechanical properties of polycarbonate/carbon nanotube/carbon fiber composites. Compos. Eng..

[54] Nishi Y., Takei H., Iwata K., Salvia M., Vautrin A. (2009). Effects of electron beam irradiation on impact value of carbon fiber reinforced thermoplastic polyetheretherketone. Mater. Trans..

[55] Nishi Y., Inoue K., Salvia M. (2006). Improvement of Charpy impact of carbon fiber reinforced polymer by low energy sheet electron beam irradiation. Mater. Trans..

[56] Park M.-S., Jung M.-J., Lee Y.-S. (2016). Significant reduction in stabilization temperature and improved mechanical/electrical properties of pitch-based carbon fibers by electron beam irradiation. J. Ind. Eng. Chem..

[57] Schlemmer B., Bandari R., Rosenkranz L., Buchmeiser M.R. (2009). Electron beam triggered, free radical polymerization-derived monolithic capillary columns for high-performance liquid chromatography. J. Chromatogr. A.

[58] Kim S.-M., Lee H.-Y., Kim S.-W. (2025). Influences of dangling bonds and van der Waals interactions on the electronic structure of tribopositive nylon polymers. Nano Energy.

[59] Nishi Y., Kitagawa S., Faudree M.C., Uchida H.T., Kanda M., Takase S., Kaneko S., Endo T., Tonegawa A., Salvia M., Kaneko S., Aono M., Pruna A., Can M., Mele P., Ertugrul M., Endo T. (2021). Improvements of strength of layered polypropylene reinforced by carbon fiber by its sizing film and electron beam under protective nitrogen gas atmosphere. Carbon Related Materials.

[60] Miura E., Uchida H.T., Okazaki T., Sagawa K., Faudree M.C., Salvia M., Kimura H., Nishi Y. (2024). Novel Treatment of 3D-Printed Short-Carbon-Fiber-Reinforced Polyamide (3D-SCFRPA66) Using Homogeneous Low-Potential Electron Beam Irradiation (HLEBI) and Ductility Enhancement. Polymers.

[61] Candau N., Chenal J.-M., Lame O., Schouwink P., Michaud V., Plummer C.J.G., Frauenrath H. (2022). Enhanced ductility in high performance polyamides due to strain-induced phase transitions. Polymer.

[62] Eiichi M., Uchida H.T., Okazaki T., Sagawa K., Satoh F., Irie H., Faudree M.C., Salvia M., Kimura H., Nishi Y. (2024). HLEBI (Homogeneous Low Potential Electron Beam Irradiation) Induced Tensile Elongation of 3D Printed Short Carbon Fiber Reinforced Polyamide (3D-SCFRPA66). Mater. Sci. Forum.

[63] Takeda K., Kimura H., Faudree M.C., Uchida H.T., Sagawa K., Miura E., Salvia M., Nishi Y. (2023). A New Strengthening Process for Carbon-Fiber-Reinforced Thermoplastic Polyphenylene Sulfide (CFRTP-PPS) Interlayered Composite by Electron Beam Irradiation to PPS Prior to Lamination Assembly and Hot Press. Materials.

[64] Kimura H., Takeda K., Uchida H.T., Faudree M.C., Sagawa K., Kaneko S., Salvia M., Nishi Y. (2022). Strengthening Process by Electron Beam to Carbon Fiber for Impact Strength Enhancement of Interlayered Thermoplastic-Polypropylene Carbon Fiber Composite. Materials.

[65] Kitagawa S., Kimura H., Uchida H.T., Faudree M.C., Kaneko S., Endoh T., Salvia M., Nishi Y. (2021). A new strengthening process of thermoplastic polypropylene reinforced by interlayered activated sizing film-free carbon fiber treated by electron beam irradiation under oxygen-rich nitrogen gas prior to assembly and hot-press. J. Compos. Mater..

[66] Nishi Y., Tsuyuki N., Uchida H.T., Faudree M.C., Sagawa K., Kanda M., Matsumura Y., Salvia M., Kimura H. (2023). Increasing Bending Strength of Polycarbonate Reinforced by Carbon Fiber Irradiated by Electron Beam. Polymers.

[67] Chen J., Czayka M., Uribe R.M. (2005). Effects of electron beam irradiations on the structure and mechanical properties of polycarbonate. Radiat. Phys. Chem..

[68] Nishi Y., Faudree M.C., Quan J., Yamazaki Y., Takahashi A., Ogawa S., Iwata K., Tonegawa A., Salvia M. (2018). Increasing Charpy Impact Value of Polycarbonate (PC) Sheets Irradiated by Electron Beam. Mater. Trans..

[69] Nathawat R., Kumar A., Kulshrestha V., Singh M., Ganesan V., Phase D.M., Vijay Y.K. (2007). Surface modification study of low energy electron beam irradiated polycarbonate film. Appl. Surf. Sci..

[70] Wang K., Pan Z., Yin L., Zhang H., Zou Y., Fei X. (2025). Study on dosimetric characteristics of polycarbonate films irradiated by electron beam. Radiat. Meas..

[71] Bansal N., Arora S. (2024). Exploring the impact of gamma rays and electron beam irradiation on physico-mechanical properties of polymers & polymer composites: A comprehensive review. Nucl. Instrum. Methods Phys. Res. Sect. B Beam Interact. Mater. Atoms..

[72] Reheem A.M.A., Atta A., Maksoud M.I.A.A. (2016). Low energy ion beam induced changes in structural and thermal properties of polycarbonate. Radiat. Phys. Chem..

[73] Rathore B.S., Gaur M.S., Singh K.S. (2011). Thermal properties of ion beam irradiated polycarbonate films. Vacuum.

[74] Radwan R.M., Abdul-Kader A.M., El-Hag Ali A. (2008). Ion bombardment induced changes in the optical and electrical properties of polycarbonate. Nucl. Instrum. Methods Phys. Res. Sect. B Beam Interact. Mater. Atoms..

[75] Technical Sheet for CF. https://shop.lab-cast.com/?pid=93479046.

[76] (1991). Testing Method for Charpy Impact Strength of Carbon Fiber Reinforced Plastics.

[77] Christenhusz R., Reimer L. (1967). Schichtdickenabhangigkeit der warmerzeugungdurch elektronenbestrahlung im energiebereich zwischen 9 und 100 keV (Layer thickness dependency of heat generation by electron irradiation in the energy range between 9 and 100 keV). Z. Angew. Phys..

[78] Zhao Y., Zhang J., Qiang S., Lu H., Li J. (2024). Effect of carbon fibers and graphite particles on mechanical properties and electrical conductivity of cement composite. J. Build. Eng..

[79] Shackelford J.F. (2000). Introduction to Materials Science for Engineers.

[80] Nishi Y., Mizutani A., Kimura A., Toriyama T., Oguri K., Tonegawa A. (2003). Effects of sheet electron beam irradiation on aircraft design stress of carbon fiber. J. Mater. Sci..

[81] Yamaguchi N., Oguri K., Tonegawa A., Nishi Y. (2004). Brittleness Control of Silica Glass Surface Irradiated by Sheet Electron Beam. Jpn. Inst. Met..

[82] Nishida T., Yasuda E. (1986). Evaluation of Dynamic Properties of Ceramics (In Japanese: Ceramics No Rikigaku Tokusei Hyouka).

[83] Weibull W. (1939). A Statistical Theory of the Strength of Materials.

[84] Weibull W. (1939). A Statistical Theory of the Strength of Materials.

[85] Quinn J.B., Quinn G.D. (2010). A practical and systematic review of Weibull statistics for reporting strengths of dental materials. Dent. Mater..

[86] Takahashi T., Morishita T., Nishi Y. (2005). Effect of Electron Beam irradiation on Stress-Strain Curves of Tensile Test of Polycarbonate Resin. Jpn. Inst. Met..

[87] Sherwood M.A. (1996). Electron Spectrosc. Relat. Phenom.

[88] Baker M.A., Bacon S.R., Sweeney S.J., Hinde S.J., Bushell A., Nunney T.S., White R.G. (2024). Femtosecond laser ablation (fs-LA) XPS—A novel XPS depth profiling technique for thin films, coatings and multi-layered structures. Appl. Surf. Sci..

[89] Yuan X., Jiang J., Wei H., Yuan C., Wang M., Zhang D., Liu L., Huang Y., Gao G.-L., Jiang Z. (2021). PAI/MXene sizing-based dual functional coating for carbon fiber/PEEK composite. Compos. Sci. Technol..

[90] Semitekolos D., Papadopoulos I., Anagnou S., Dashtbozorg B., Li X., Dong H., Charitidis C.A. (2024). Nanomaterial-Enhanced Sizings: Design and Optimisation of a Pilot-Scale Fibre Sizing Line. Fibers.

[91] Singh S., Mai P., Borowiec J., Zhang Y., Lei Y., Schober A. (2018). Donor–acceptor Stenhouse adduct-grafted polycarbonate surfaces: Selectivity of the reaction for secondary amine on surface. Roy. Soc. Open Sci..

[92] Faudree M.C., Nishi Y., Gruskiewicz M. (2012). Effects of electron beam irradiation on Charpy impact value of short glass fiber (GFRP) samples with random distribution of solidification texture angles from zero to 90 degrees. Mater. Trans..

